# Biomolecular regulation, composition and nanoarchitecture of bone mineral

**DOI:** 10.1038/s41598-018-19253-w

**Published:** 2018-01-19

**Authors:** Atharva A. Poundarik, Adele Boskey, Caren Gundberg, Deepak Vashishth

**Affiliations:** 1 Department of Biomedical Engineering, Center for Biotechnology and Interdisciplinary Studies, Rensselaer Polytechnic Institute, Troy, NY 12180 USA; 2 Musculoskeletal Integrity Program, Hospital for Special Surgery, New York, NY, 10021 USA; 3 Department of Orthopedics and Rehabilitation, Yale University, New Haven, CT, 06520 USA

## Abstract

Tough natural nanocomposites like bone, nacre and sea sponges contain within their hierarchy, a mineral (phosphate, silicate or carbonate) phase that interacts with an organic phase. In bone, the role of mineral ultrastructure (organization, morphology, composition) is crucial to the mechanical and biological properties of the tissue. Better understanding of mineral interaction with the organic matrix, in particular non-collagenous proteins, osteocalcin (OC) and osteopontin (OPN), can lead to better design of biomimetic materials. Using small angle x-ray scattering (SAXS) and wavelength dispersive spectroscopy (WDS) on single (OC^−/−^ and OPN^−/−^) and double (OC-OPN^−/−;−/−^) genetic knockout mice bones, we demonstrate that both osteocalcin and osteopontin have specific roles in the biomolecular regulation of mineral in bone and together they are major determinants of the quality of bone mineral. Specifically, for the first time, we show that proteins osteocalcin and osteopontin regulate bone mineral crystal size and organization in a codependent manner, while they independently determine crystal shape. We found that OC is more dominant in the regulation of the physical properties of bone mineral, while OPN is more dominant in the regulation of the mineral composition.

## Introduction

Tough natural nanocomposites like bone, dentin, nacre^1^ and sea sponges^2^ contain within their hierarchy, a mineral (phosphate, silicate or carbonate) phase^3^. The regulation of mineral ultrastructure is various biological systems is a complex biophysical process. Mineralization in various natural nanocomposites is affected by the coordinated action of various extracellular organic molecules, including proteins, on the inorganic phase. Similar to these nanocomposites, ultra-structural composition and organization of the mineral phase in bone is critical for its enhanced toughness^4^. For example, bone diseases that increase bone’s propensity to fracture, such as osteogenesis imperfecta^5,6^ and osteopetrosis^7^, are known disturb the nanoscale heterogeneity in bone matrix by altering mineral crystal thickness and organization.

Bone mineral crystals are primarily constituted of hydroxyapatite. The crystals are present within the gap regions^8^ of the collagen fibril and in the extrafibrillar regions (space located outside the collagen fibril)^9^, and each has a thickness of up to 5 nm^10^. Bone crystals exhibit a plate or needle-like morphology and are aligned such that their c-axis lies along the long axis of collagen fibrils^8^. They vary in crystallinity (crystal size and perfection) due to trace impurities including trace element ions like carbonate (CO_3_^2−^), fluoride (F^−^), sodium (Na^+^) and magnesium (Mg^2+^) ions as well as vacancies. The impurities are incorporated as substitutions within the HA lattice^11^. While cations like Mg^2+^ and Na^+^ substitute for Ca^2+^ sites, anions like F^−^ substitute the OH^−^ sites within the HA lattice, modulating hydrogen bonding. Carbonate substitutes for both hydroxyl (B-type substitution) and phosphate (A-type substitution). Trace elements are important constituents of bone mineral. The amount of sulfur (S), also present in trace quantities, has been correlated with proteoglycan content present in bone matrix^12^. Trace elements such as F and Mg, significantly impact bone metabolism. Fluorine, for example, induces bone formation through increased osteoblastic activity but inhibits mineralization^13^. Magnesium substitution stimulates osteoblast proliferation and its depletion causes bone fragility and bone loss^14^.

Non-collagenous proteins (NCPs) including osteocalcin (OC) and osteopontin (OPN) have long been associated with regulating bone mineral. Osteocalcin, a protein predominantly synthesized by osteoblasts, is known for its strong binding affinity to bone mineral^15^. OC’s mineral binding capacity is facilitated by its carboxyglutamic acid residues providing a function that has been well conserved through evolution^16^. Osteopontin (meaning ‘bone bridge’), a large highly charged glycoprotein, also binds to extracellular calcium via its acidic (aspartate and glutamate) and phosphorylated (serine and threonine) residues and plays a role in nucleating bone mineral^17^. Here, we ask whether OC and OPN regulate crystal size, morphology and composition of bone at the nanoscale. If so, how do they act? And, do they act through independent or codependent functional motifs?

In previous studies, the use of genetically engineered mice has allowed for a careful evaluation of the role of NCPs in regulation of mineralization in bone. Single knockout mice, deficient in osteocalcin (OC^−/−^)^18^ and osteopontin (OPN^−/−^)^19^, reveal that OC and OPN influence the material composition of mineral crystals. Fourier transform infrared microscopy (FTIRM) demonstrates that bones of mice lacking osteocalcin, reported to be an inhibitor of bone formation^18^, contain smaller mineral crystals with lower carbonate substitution^20^. *In vitro*, highly phosphorylated osteopontin can nucleate, facilitate and inhibit crystal growth, while less phosphorylated OPN inhibits HA growth^17^. Although, OPN^−/−^ mice bones do not show distinct morphological differences as compared to WT bones^19^, OPN^−/−^ bones show greater mineral to matrix ratio and increased crystal maturity in comparison with WT^21^ suggesting that OPN may influence bone’s nanostructure via physicochemical pathways.

Single knockout mice models (for example OPN^−/−^ or OC^−/−^) alone fail to elucidate the specific roles of NCPs in the regulation of bone mineral properties as the loss of one NCP may be compensated by the other – such redundancy is often seen in key biological processes^22^. The OC-OPN^−/−;−/−^ genotype, that lacks both OC and OPN, was generated by breeding OC^−/−^ mice^18^ with OPN^−/−^ mice^19^. The absence of both OC and OPN from these mice allows us to investigate the independent and redundant roles of OC and OPN in the bone mineralization process.

Here, we use this mouse genotype (OC-OPN^−/−;−/−^) as well as single knockouts (OPN^−/−^ or OC^−/−^) to elucidate the individual roles of OC and OPN in bone mineral regulation. Through the use of small angle x-ray scattering (SAXS), a technique that allows investigation of length scales from 0.5–50 nm^23^, OC-OPN^−/−;−/−^ mineral characteristics were compared to both WT and single knock-outs (OC^−/−^ and OPN^−/−^). Wavelength dispersive spectroscopy (WDS) was used to obtain the elemental composition of each genotype including calcium to phosphorus molar ratios. Scanning electron microscopy (SEM) was employed to qualitatively examine nanoscale structural changes that occur in bone mineral crystals in the absence of osteocalcin and/or osteopontin.

## Results

**Figure 1:**
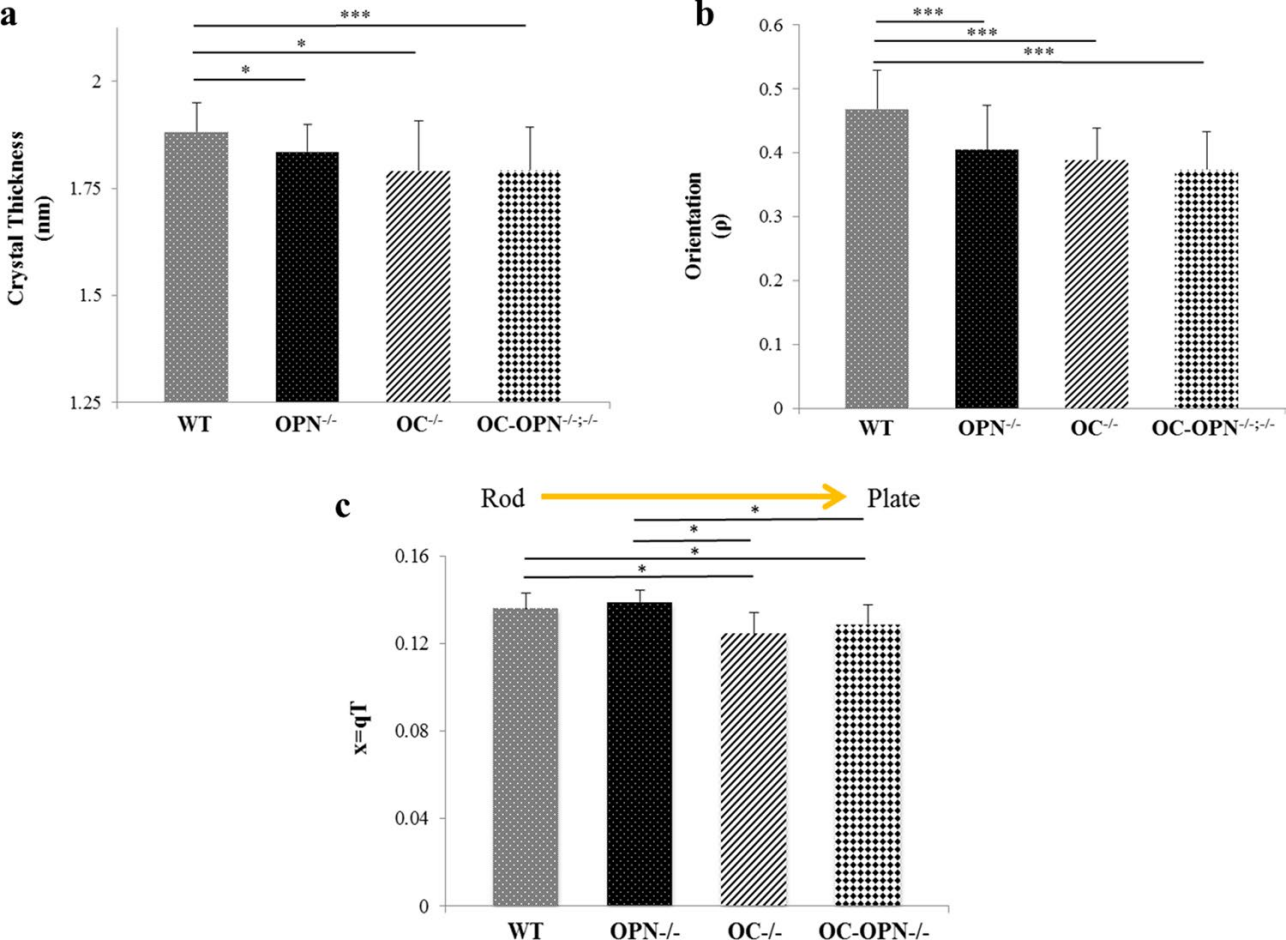
(**a**) Mineral crystal thickness for all genotypes (* and ** denote p < 0.05 and p < 0.01 respectively as determined by ANOVA post-hoc SNK test). No differences were observed between the knock-out groups. (**b**) Mineral crystal orientation for all genotypes. (*** denotes p < 0.001 as determined by ANOVA post hoc SNK test). If ρ = 0, the crystals have no predominant orientation within the plane of the section. As ρ increases, crystals are increasingly aligned along the collagen fibril; ρ = 1 implies all crystals are aligned along the principal axis (fibril direction). (**c**) Values of x for all four genotypes. Shape changes from rod-like to plate-like as shown. Values of x for rod-like WT and OPN^−/−^ are significantly different (p < 0.05, One-way ANOVA post hoc SNK test) from those of plate-like OC^−/−^ and OC-OPN^−/−;−/−^.

Quantitative analysis of 2D SAXS spectra revealed that the absence of either OC and/or OPN reduced crystal thickness (Fig. 1a) and reduced crystal orientation (Fig. 1b) in the OC^−/−^, OPN^−/−^ and OC-OPN^−/−;−/−^ mice bones. Crystals in mice bones, devoid of OC and OPN, were smaller and less aligned along the long axis of collagen (and less anisotropic or less heterogeneous) than WT (p < 0.05). Crystal thickness of the WT group (1.88 ± 0.07 nm) was greater than that of OPN^−/−^ (1.83 ± 0.07 nm), OC^−/−^ (1.79 ± 0.12 nm) and OC-OPN^−/−;−/−^ (1.79 ± 0.10 nm) groups (p < 0.05). Degree of orientation of crystals (ρ) in WT group (0.47 ± 0.06) was also greater than that in OPN^−/−^ (0.41 ± 0.07), OC^−/−^ (0.39 ± 0.05) and OC-OPN^−/−;−/−^ (0.37 ± 0.06) groups (p < 0.05).

We performed Spearman correlation analyses between crystal thickness and orientation. Results confirmed our hypothesis that OC and OPN are codependent in the regulation of crystal thickness (variable x) and orientation (variable y). Thickness correlated positively with orientation suggesting that thicker (and thus more mature) WT crystals (R = 0.632, p < 0.001) were more longitudinally aligned. Therefore, the presence of both OC and OPN, increased crystal maturity and led to better alignment. Additionally, no correlation was seen between thickness and orientation in OC^−/−^, OPN^−/−^ and OC-OPN^−/−;−/−^ mice (p > 0.05). Hence, the absence of either or both proteins disrupts the interdependent relationship between mineral crystal thickness and crystal orientation as seen in WT mice bone. In contrast to the interdependence seen in the regulation of crystal thickness and orientation, we found that OC and OPN independently affected crystal shape (Fig. 1c).

**Figure 2:**
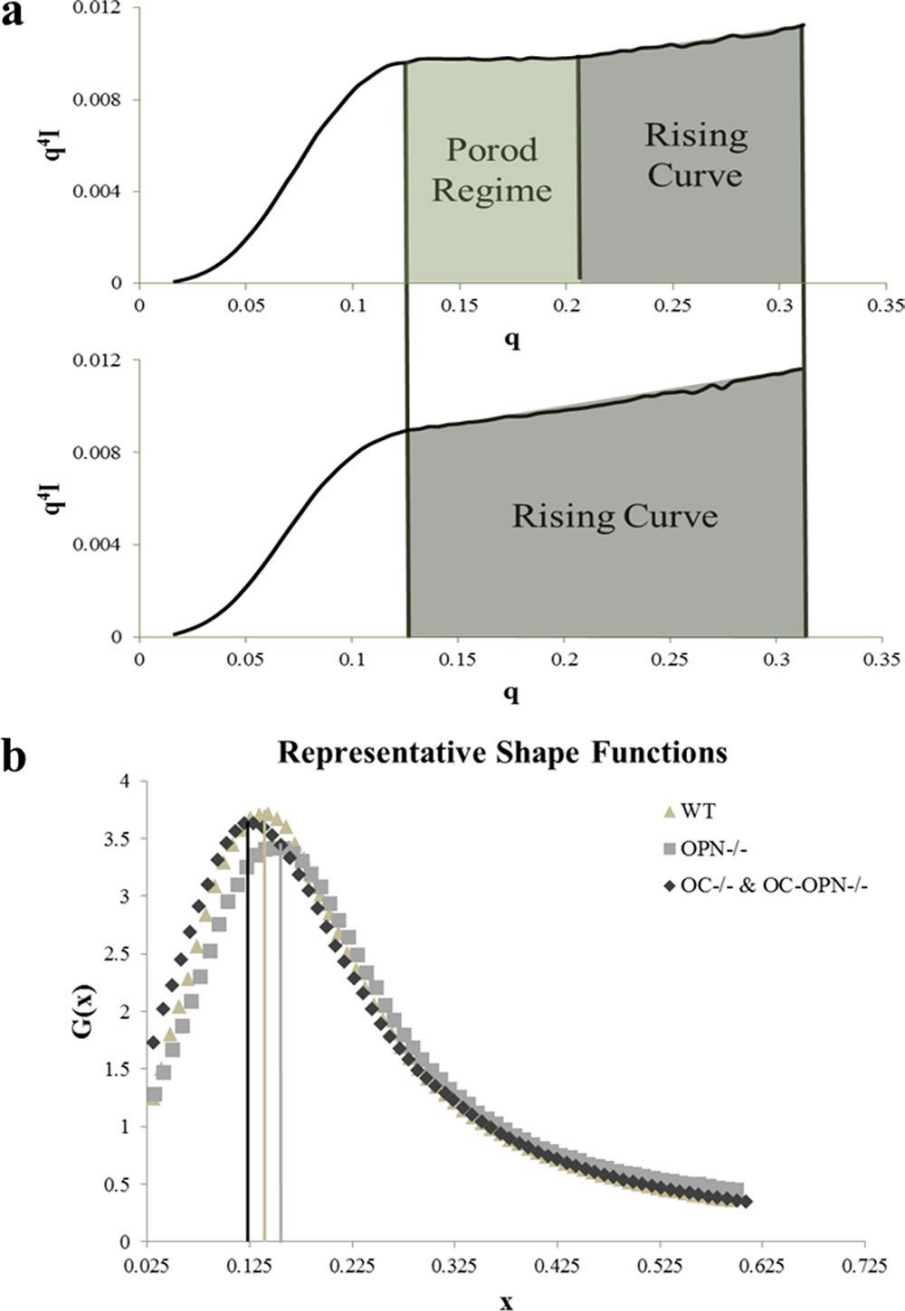
(**a**) Representative plots showing the Porod regime for the WT bone (top) and a deviation from Porod’s law for the double knock-out bones (bottom). (**b**) The Kratky curve shifts to the right in the OPN^−/−^ group indicating rod-like crystals. In the OC^−/−^ and OC-OPN^−/−;−/−^ groups, there is a shift to the left, indicating more plate-like crystals as compared to WT.

Qualitative analysis of Porod plots **(**Fig. 2a**)** revealed more details on the ultrastructure of bone mineral in each genotype. In contrast to WT and OPN^−/−^, that display a characteristic plateau (or Porod’s region) associated with sharp interfaces between the organic and inorganic phases, OC^−/−^ and OC-OPN^−/−;−/−^ display a rising curve. The absence of Porod’s region in mice devoid of osteocalcin suggests a loss of order at lengths below the crystal dimensions. Such loss is characterized by loose mineral aggregates with ill-defined surface boundaries and broken interfaces. The Kratky curve shifted to the left (as represented by lowered x, where x ( = qT) is a composite function of q, the scattering vector and T, crystal thickness (additional details in Methods), in the OC^−/−^ and OC-OPN^−/−;−/−^ genotypes as compared to WT; in OPN^−/−^ the curve shifted to the right (as represented by higher x values) (Fig. 2b). The normalized q values for the WT (0.136 ± 0.007) and OPN^−/−^ (0.139 ± 0.005) mice were greater than those for OC^−/−^ (0.124 ± 0.010) and OC-OPN^−/−;−/−^ (0.129 ± 0.009) mice (p < 0.05). Thus, crystal shape in OC^−/−^ and OC-OPN^−/−;−/−^ mice bone deviates to a more plate-like form from a rod-like form^24^. Spearman’s correlation analysis shows that OC and OPN affect crystal shape differently. In the absence of OC (OC^−/−^), crystal thickness, T, correlates positively (R = 0.702, p < 0.001) with x (independent shape function variable, x = qT), indicating that thicker crystals are more rod-like. In the absence of OPN (OPN^−/−^), T correlates negatively with x (R = −0.330, p = 0.074) indicating thicker crystals are more plate-like.

**Figure 3:**
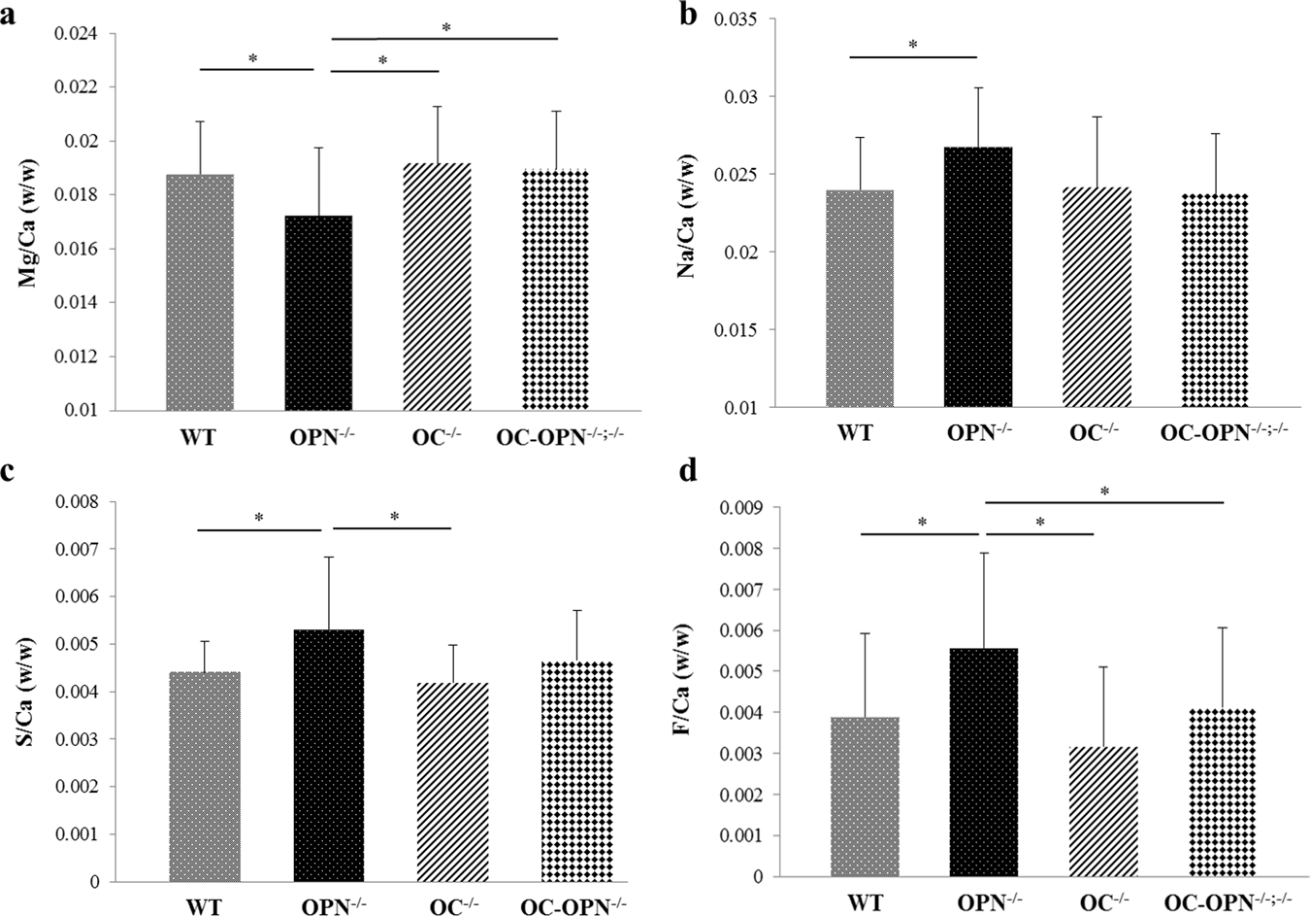
Trace element levels as measured per unit of Ca (w/w), for the elements, Mg **(a)**, Na **(b)**, S **(c)** and F **(d)**.

Quantitative WDS analyses revealed significant differences in trace element levels present in the bone matrix of the four genotypes investigated (Fig. 3). Mg/Ca (w/w) ratio was lower (One-way ANOVA post hoc SNK test) in OPN^−/−^ group (0.0172 ± 0.0025) as compared to WT (0.0188 ± 0.0020), OC^−/−^ (0.0192 ± 0.0021) and OC-OPN^−/−;−/−^ (0.0189 ± 0.0022) groups (Fig. 3a). Na/Ca (w/w) ratio was higher in the OPN^−/−^ group as compared to the WT group (p < 0.05, Student’s t-test). Na content in the OC^−/−^ and OC-OPN^−/−;−/−^ bones was similar to that in WT (Fig. 3b). S/Ca (w/w) ratio was higher in bones of OPN^−/−^ mice (0.0053 ± 0.0015) as compared to WT (0.0044 ± 0.0006), OC^−/−^ (0.0042 ± 0.0008) and OC-OPN^−/−;−/−^ (0.0046 ± 0.0010, p = 0.054) mice bones (ANOVA post hoc SNK test) (Fig. 3c). F/Ca (w/w) ratio was higher (One-way ANOVA post hoc SNK test) in the OPN^−/−^ group (0.0056 ± 0.0023) as compared to WT (0.0039 ± 0.0020), OC^−/−^ (0.0032 ± 0.0019) and OC-OPN^−/−;−/−^ (0.0041 ± 0.0019) groups (Fig. 3d).

In particular, OPN^−/−^ mice showed reduced levels of Mg (p = 0.023) and increased levels of S (p = 0.007), F (p < 0.003), Na (p = 0.054) and as compared to the WT, OC^−/−^ and OC-OPN^−/−;−/−^ genotypes. Surprisingly, the levels of these elements were similar in the WT, OC^−/−^ and OC-OPN^−/−;−/−^ genotypes, indicating that OPN may be a dominant regulator of crystal composition as compared to OC and other matrix NCPs. No significant differences were seen amongst the groups in the Ca/P ratio, with one-way ANOVA. However, Student’s t-tests showed increase in Ca/P molar ratio in OC^−/−^ (1.442 ± 0.064, p = 0.028) and OPN^−/−^ (1.443 ± 0.086, p = 0.064) groups as compared to WT (1.404 ± 0.060). The Ca/P molar ratio for OC-OPN^−/−;−/−^ mice bones (1.428 ± 0.070) was not significantly different from WT.

**Figure 4:**
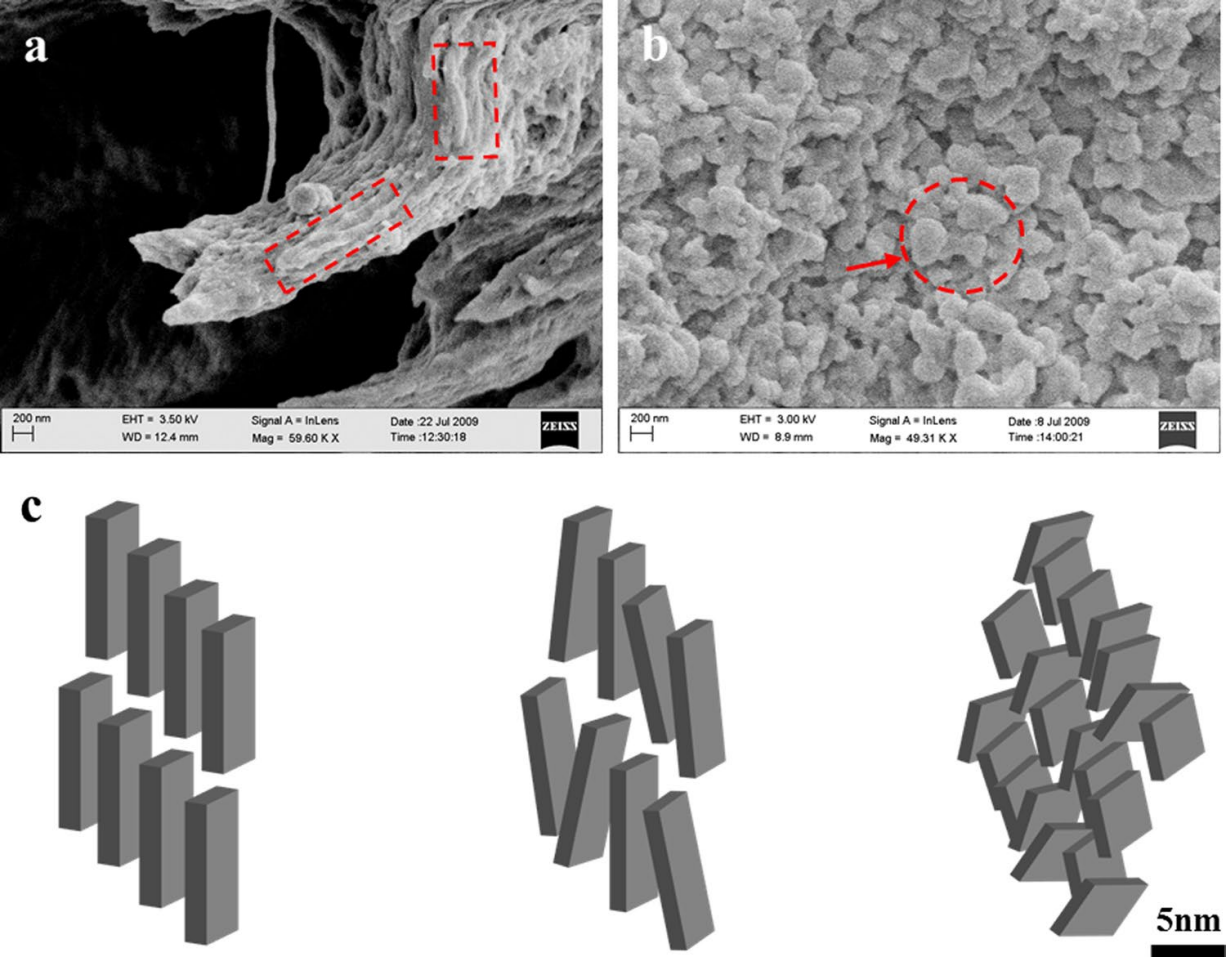
High magnification SEM images of (**a**) WT and (**b**) OC-OPN^−/−;−/−^ mice showing mineral organization. (**c**) Schematic of crystal arrangement in WT (left), OPN^−/−^ (center) and OC^−/−^, OC-OPN^−/−;−/−^ (right).

A scanning electron microscope (SEM) analysis of the WT and OC-OPN^−/−;−/−^ bones illustrates that the mineral in WT bone is rod-like (Fig. 4a, indicated by dotted rectangular outline in red), and is oriented in the direction of the collagen fibril. In contrast, OC-OPN^−/−;−/−^ mice bones exhibit rounded, plate-like crystals (Fig. 4b, arrow) which appear to link together to form aggregates (Fig. 4b, circled). The SEM characterization corroborates our SAXS findings detailed above. A schematic (Fig. 4c) illustrates mineral crystal morphology and arrangement in each of the genotypes studied.

## Discussion

NCPs are secreted during osteoid mineralization and their physico-chemical interactions with bone mineral have been well documented *in vitro*^17^. This study demonstrates for the first time, that OC and OPN have multiple functions in the regulation of bone mineral properties. Through the use of both, single and double knock-out mice genotypes, the results presented show that the absence of either or both NCPs, OC and OPN, from bone matrix significantly reduces crystal thickness and alters crystal shape as compared to WT controls. Furthermore, loss of NCPs OC and OPN from the matrix disrupts the longitudinal crystal orientation and changes trace elemental composition of mineral crystals. Whilst OC is more dominant in the regulation of the physical properties of bone mineral such as size (mineral thickness), OPN is more dominant in the regulation of trace elemental composition of bone mineral.

Osteocalcin serves primarily to ensure growth of crystals. Existence of such a function for OC in bone can also be supported by previous *in vitro* observations that show precise arrangement of Ca^2+^ sites on the hydroxyapatite surface lattice through controlled apposition of Ca^2+^, OH^−^ and PO_4_^3−^ ions by γ-carboxyglutamic acid residues (Gla)] of OC^15^. The smaller and imperfect crystals observed in the OC^−/−^ mice, can be explained by poorly regulated mineral apposition (i.e. incomplete and incoherent) leading to the formation of aggregates (Fig. 4b). Thus, OC may function to maintain bone anisotropy by preventing the formation of aggregates. Furthermore due to an overall reduction of organized interfacial interaction, as is likely in planar crystals, mineral aggregation can limit mineral interaction with the organic components and reduce the efficacy of load transfer through the matrix and bone toughness^25^.

Unlike OC, which is a more dominant regulator of crystal size and morphology, here we show that OPN principally functions as a nucleator of mineral in bone and regulates crystal composition and crystal growth. In particular, we found a significant reduction in the crystal thickness of OPN^−/−^ genotype as compared to WT (Fig. 1a) demonstrating that the presence of OPN is necessary for normal crystal growth. OPN can transiently bind to collagen and serve as a nucleation scaffold for bone mineral during the early stages of mineralization. This process is likely mediated by phosphorylated residues present on OPN^26^. Sufficient PO_4_^3−^ groups can encourage the assembly of a critical HA nucleus size for crystal growth and also allow the accumulation of enough Ca^2+^ and Mg^2+^ ions to attract OC Gla groups.

OC also binds with great affinity to Mg^27^ and our results indicate that OC may be involved in the removal of Mg from mineral because OC controls crystal growth during the mineralization process. The trace element sulfur is present in larger amounts within osteoid and has been shown to vary directly with the amount of chondroitin sulfate^12^. It likely that there is presence of increased levels of negatively charged sulfonated glycosaminoglycans within OPN^−/−^ mice bone as compared to the other genotypes^12^. An increase in the content of sodium in OPN^−/−^ bones, could be due to increased chondroitin sulfate levels. Consistent with the above concept, we found that OPN is a dominant regulator of crystal chemical composition as compared to OC.

Since OPN is a phosphorylated protein, it may have an integral role in the substitution of phosphate ions within bone mineral^28^. Thus, the increased Ca/P molar ratio in OPN^−/−^ mice can be explained by the loss of phosphate groups in these mice. The substitution of CO_3_^2−^ and HPO_4_^2−^ for PO_4_^3−^ sites within the HA lattice^20^ may therefore control crystal maturity and alter the Ca/P molar ratio in the OC^−/−^ mice bones as was found in this study. It is noteworthy that these results differ from those reported in previous FTIRM studies on ovariectomized (OVX) WT and OC^−/−^ mice^20^ showing a reduced Ca/P molar ratio in OC^−/−^ compared to WT mice. This discrepancy between our and previous studies is explained as follows. Accelerated turnover in OVX WT mice prevents crystal maturation which is known to correlate with increased size^20^. Such changes may result in an apparent increase in the Ca/P value in OC^−/−^ OVX mice which most likely contain a more uniform distribution of small, immature crystals as opposed to a combination of mature and immature crystals in 6-month old male mice bones used here.

It is also known that the interaction of OC with Ca^2+^ and with OPN and Ca^+2^ and collagen, causes conformational changes that facilitate OC attachment onto the hydroxyapatite surface^15,17^. The continuous phosphorylation and dephosphorylation of OPN is capable of modulating the local environment by regulating the flux of phosphate groups into bone mineral. The above process is likely enhanced in the WT crystal growth, where both OC and OPN are present. Alternatively, OPN’s ability to interact directly with OC^29^ may explain its role in crystal growth. Such interdependence between OC and OPN could be a principal mechanism through which crystal thickness is regulated. Indeed, *in vitro* experiments have shown that crystal growth is enhanced in the presence of both OC and OPN^17^. Our results show that in the absence of either one, or both, crystal growth and arrangement deviates from WT control.

In particular, we show that OC partially rescues crystal thickness (Fig. 1a) in the OPN^−/−^ mice. However, the similarity in crystal shape and crystal thickness values of OC^−/−^ and OC-OPN^−/−;−/−^ indicates that OPN is unable to mitigate the effect that the loss of OC has on crystal shape and thickness. No differences in the trace elements were noted between the WT, OC^−/−^ and OC-OPN^−/−;−/−^ (Fig. 3). Therefore we note the presence of redundancies in the regulation of crystal composition where an imbalance in the OPN^−/−^ mice is mitigated by an alternative mechanism associated with simultaneous loss of OC and OPN in the OC-OPN^−/−;−/−^ mice. We postulate the involvement of other NCPs, for example proteoglycans and DMP-1, in such functional redundancies. Also, the physicochemical processes may be mediated by cells (osteoblasts or osteoclasts) in the presence/absence of NCPs. However, it is important to note that even in the absence of OC and OPN, there is formation of mineral crystals with certain size, shape and orientation. Consequently while redundancies may exist, the matrix nano-architecture is certainly not optimal. In that regard, the effect of absence of other NCPs on mineral properties needs further examination.

**Figure 5:**
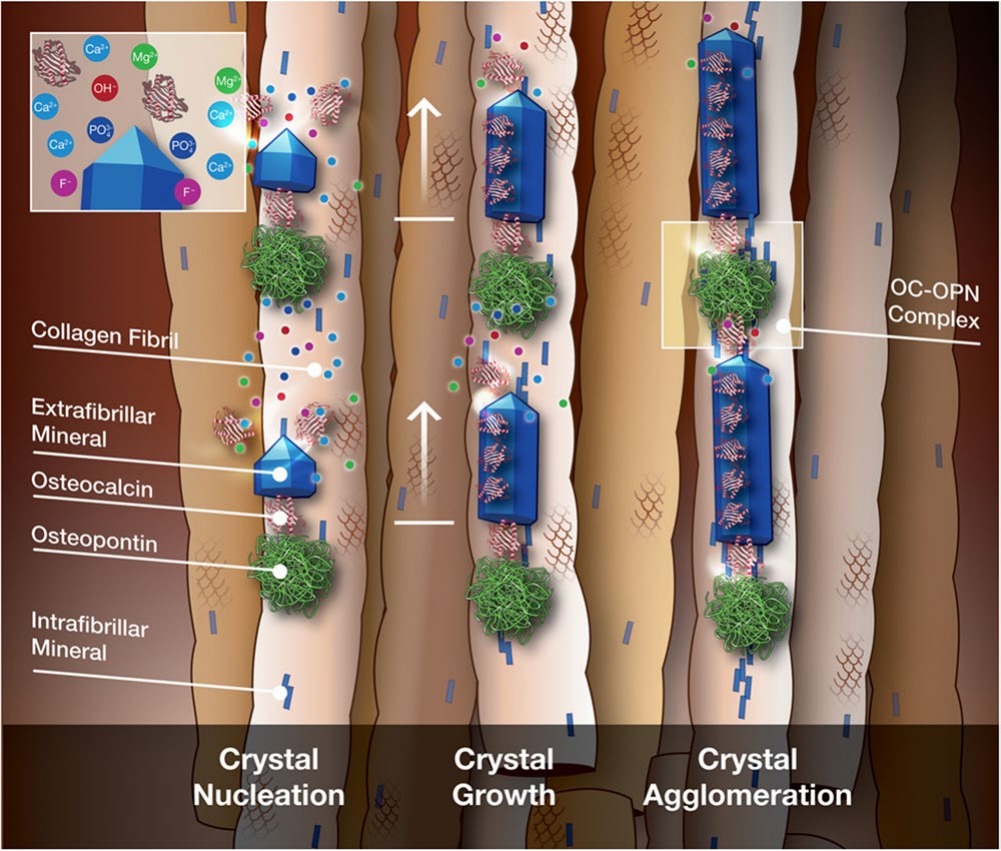
Mechanism of biomolecular regulation of mineral in bone. Crystals nucleate (left) under the cooperative action of OC and OPN, and possibly other NCPs. The highly charged OPN aids in the recruitment of essential ionic groups and charged trace elements. Proteoglycans or ion-transport mechanisms may also be responsible for such a recruitment process. OC regulates crystal growth (center) along the long axis of collagen, by attaching at favorable crystallographic locations on the growing crystal. Finally, crystals agglomerate with each other through the formation of OC-OPN linkages, when they arrive at another nucleation site. (Image credits: Mark Esposito, 2017).

Based on the above results, a new model (Fig. 5) on the mechanistic roles of OC and OPN in bioregulation of crystal growth is proposed. Per our model, mineral crystals nucleate on the collagen fibril with the cooperative action of OC and OPN. Subsequent action by OC leads to regulated crystal growth along the collagen fibril in order to maximize mineral organic interaction. OC recruits and arranges constituent hydroxyapatite ions, principally Ca^2+^ and PO_4_^−3^ onto the growing mineral crystals. OC attaches to Ca^2+^ on the crystal surface to ensure that the crystal shape and size are maintained. OPN assists in the recruitment of other ionic species like Mg^2+^ that substitute for Ca^2+^ within the HA lattice and also control the influx of negatively charged groups like F^−^ during the crystal growth phase. Crystals then conjugate with other crystals through the formation of OC-OPN linkages. The interfacial OC-OPN linkages, connecting the organic and mineral components of bone matrix, are crucial in determining bone matrix properties^25^. Any disruption in the bone nanostructure due to absence of OC or OPN hinders bioregulation of mineral in bone.

The mineral phase is crucial for the load bearing function of bones^3^. The conservation of γ-carboxyglutamic acid sequences in OC^30^ as well as phosphorylation sequences and negatively charged amino acid groups (aspartate/glutamate) in OPN^31^ throughout evolution highlight the significance of negatively charged groups in the maintenance of bone mineral. Findings of this study demonstrate, for the first time, that OC and OPN have multiple functional roles in the bioregulation of bone mineral. In addition to being more dominant in the control of crystal size and morphology than OPN, OC also mitigates the effects that the absence of OPN has on mineral crystal characteristics. In contrast, OPN, the highly charged and phosphorylated protein, is more dominant than OC in regulating trace element crystal composition in bone mineral. The role of OC and OPN presented here, gives insight into how nature can regulate nanoscale material properties of other mineralized materials (nacre, abalone, etc.).

## Materials and Methods

### Mice Models

Four mice genotypes including wild-type (WT), osteocalcin knock-out (OC^−/−^), osteopontin knock-out (OPN^−/−^) and osteocalcin-osteopontin double knock-out (OC-OPN^−/−;−/−^) were used in this study. The WT mice were generated from heterozygote crosses of OC-OPN^−/−;−/−^ mice. Male or female mice aged six months (n = 5 for each genotype) were sacrificed and mid-diaphyseal cross-sections of their femora were analyzed. The bones were dehydrated in 70% and 100% ethanol prior to SAXS, WDS or SEM analyses. All methods were carried out in accordance with relevant guidelines and regulations. All animal experimental protocols were approved by IACUC committees at Yale and RPI.

### Small angle x-ray scattering

SAXS employs elastic scattering of x-rays to analyze nanoscale features in materials. Evaluation of relationships between scattered intensity (I), scattering vector (q) and azimuthal angle (ψ) allow estimation of thickness, orientation and shape of mineral crystals in bone; the scattering vector q is given by 4sin(θ)/λ (λ is the wavelength of x-rays (0.154 nm) and θ is the angle between the x-ray beam and detector)^23^.

2-D SAXS spectra were acquired on a Bruker Nanostar-U (Bruker, Switzerland) with a rotating anode, turbo copper x-ray source and a noise-free Hi-STAR 2-D detector with real-time photon counting ability. Spectra were acquired at selected points (n = 10) for each mouse bone specimen. Using Porod’s law (P = Iq^4^), the Porod constant P was calculated for every 2-D spectrum from regions (Porod regime) where Iq^4^ was constant. In cases where Iq^4^ steadily increased (rising curve regime), the slope’s median Iq^4^ value was used. The Porod law allows for the calculation of surface to volume ratio, or thickness of the mineral particles, by considering bone as a two-phase material of mineral and organic phases where the mineral-organic interface is sharp^32^. Deviations from Porod’s law can be interpreted to reveal information about the material organization.

Kratky plots (q^2^I vs. q), were used to evaluate the area under the Kratky curve (J). Similar to Porod’s constant ‘P’, the parameter J is an invariant and used in the computation of crystal thickness (4 J/πP)^10^. To determine the average shape of mineral crystals, we analyzed the Kratky plots based on previous studies^10,24^. By defining a new variable x = qT (normalized q) and a new function G(x) = q^2^I(q)/I(0), the Kratky function was normalized with respect to thickness. The rescaled G(x) function defines only the shape of the crystals irrespective of the thickness. Orientation of bone mineral crystals (ρ) in the plane of the longitudinal cross-section was computed from I(ψ) versus ψ (0 < ψ < 2π) plots^33^, where ψ is the azimuthal angle within the plane of the 2-D spectrum.

### Wavelength Dispersive Spectroscopy (WDS)

WDS was performed on a Cameca SX-10 Electron Microprobe Analyzer (Cameca, France), equipped with five crystal spectrometers, secondary electron (SE) and back-scattered electron (BSE) detectors and a Bruker AXS Quantax 200 EDS system. The large area crystals (four LPET, two LLIF, one LPCO), used for the WDS, were housed in four of the five spectrometers. WDS allows quantitative analyses of multiple elements with a high degree of precision (ppm). As opposed to energy dispersive x-ray spectroscopy (EDXS), WDS allows separation of elemental peaks that are otherwise close and indistinguishable within the EDXS spectrum. For each genotype, an average of all specimens was taken (n = 5 specimens per group). For each specimen, 10 data acquisition points were obtained. Samples for analysis were 100 μm thick mid-diaphyseal cortical bone sections coated with ~30 nm layer of carbon prior to analyses. An operating voltage of 15 kV and an electron beam spot size of 10 μm were used for all measurements. For all specimen, data was acquired for the following elements: calcium (Ca), phosphorous (P), magnesium (Mg), fluorine (F), sodium (Na) and sulfur (S). Prior to data acquisition, points of interest were randomly selected on backscattered electron microscopy images obtained on an SEM affixed to the microprobe.

### Scanning electron microscopy

SAXS findings were validated using scanning electron microscopy on a Carl Zeiss Supra SEM (Carl Zeiss Microscopy, Thornwood, U.S.A.) equipped with InLens SE detector, Everhart-Thornley SE detector and a Robinson BSE detector, with voltage and current limits of 30 kV and 20 nA, respectively. Mice bone were fractured in three-point bending. Fractured diaphyseal regions of WT, OC^−/−^, OPN^−/−^ and OC-OPN^−/−;−/−^ bones (n = 3 each) were coated with ~30 nm thick layer of platinum and the fracture surfaces were imaged using an InLens detector at 2.5–5 kV. Images were taken at magnifications of ~50,000× to identify morphology of bone mineral present in the four genotypes.

### Statistical Analyses

All analyses were done on SSPS 11.0 software using one-way ANOVA and post-hoc Students-Neumann-Keul tests. A confidence level of p < 0.05 implied statistical significance between the groups. Results were reported as mean ± standard deviation with *, ** and *** denoting p < 0.05, p < 0.01 and p < 0.001 respectively. In case of absence of significance with ANOVA, individual groups were subjected to the Student’s t-test to identify difference between a specific knock-out and WT control. Spearman correlation analyses were also done for crystal thickness, crystal orientation and crystal shape parameters.

### Data Availability

The datasets generated during and/or analysed during the current study are available from the corresponding author on reasonable request.

## References

[1] Li X, Chang WC, Chao YJ, Wang R, Chang M (2004). Nanoscale structural and mechanical characterization of a natural nanocomposite material: The shell of red abalone. Nano Lett..

[2] Aizenberg J (2005). Skeleton of Euplectella sp.: Structural hierarchy from the nanoscale to the macroscale. Science.

[3] Currey JD (2005). Hierarchies in biomineral structures. Science.

[4] Tai K, Dao M, Suresh S, Palazoglu A, Ortiz C (2007). Nanoscale heterogeneity promotes energy dissipation in bone. Nature Materials.

[5] Fratzl P, Paris O, Klaushofer K, Landis WJ (1996). Bone mineralization in an osteogenesis imperfecta mouse model studied by small-angle x-ray scattering. J Clin Invest..

[6] Vetter U, Eanes ED, Kopp JB, Termine JD, Gehron Robey P (1991). Changes in apatite crystal size in bones of patients with osteogenesis imperfecta. Calcified Tissue International.

[7] Boskey AL, Marks SC (1985). Mineral and matrix alterations in the bones of incisors-absent (ia/ia) osteopetrotic rats. Calcified Tissue International.

[8] Landis WJ, Song MJ, Leith A, McEwen L, McEwen BF (1993). Mineral and organic matrix interaction in normally calcifying tendon visualized in three dimensions by high-voltage electron microscopic tomography and by graphic image reconstruction. J Struct Biol..

[9] McNally EA, Schwarcz HP, Botton GA, Arsenault AL (2012). A Model for the Ultrastructure of Bone Based on Electron Microscopy of Ion-Milled Sections. PLoS One.

[10] Fratzl P, Schreiber S, Klaushofer K (1996). Bone Mineralization as Studied by Small-Angle X-Ray Scattering. Connective Tissue Research.

[11] Kuhn-Spearing, L., Rey, C., Kim, H. M.& Glimcher, M. J. Carbonated apatite nanocrystals of bone. *In Synthesis and processing of nanocrystalline powder: Proceedings of a Symposium Cosponsored by the Materials Design and Manufacturing Division (MDMD), Power Metallurgy Committee of the Minerals, Metals & Materials Society, and the Federation of European Materials Societies* (Editor: David Lee Bourell), 57–68 (1996).

[12] Schaffler MB, Burr DB, Frederickson RG (1987). Morphology of the Osteonal Cement Line in Human Bone. The Anatomical Record.

[13] Shephard JH, Shephard DV, Best SM (2012). Substituted hydroxyapatites for bone repair. Mater Sci: Mater Med.

[14] Laurencin D (2011). Magnesium Incorporation into Hydroxyapatite. Biomaterials.

[15] Dowd TL, Rosen JF, Li L, Gundberg CM (2003). The Three-Dimensional Structure of Bovine Calcium Ion-Bound Osteocalcin Using 1H NMR Spectroscopy. Biochemistry.

[16] Laizé V, Martel P, Viegas CS, Price PA, Cancela ML (2005). Evolution of matrix and bone gamma-carboxyglutamic acid proteins in vertebrates. Journal of Biological Chemistry.

[17] Gericke A (2005). Importance of Phosphorylation for Osteopontin Regulation of Biomineralization. Calcified Tissue International.

[18] Ducy P (1996). Increased bone formation in osteocalcin-deficient mice. Nature.

[19] Rittling SR (1998). Mice Lacking Osteopontin Show Normal Development and Bone Structure but Display Altered Osteoclast Formation *In Vitro*. Journal of Bone and Mineral Research.

[20] Boskey AL (1998). Fourier Transform Infrared Microspectroscopic Analysis of Bones of Osteocalcin-Deficient Mice Provides Insight Into the Function of Osteocalcin. Bone.

[21] Boskey AL, Spevak L, Paschalis E, Doty SB, McKee MD (2002). Osteopontin Deficiency Increases Mineral Content and Mineral Crystallinity in Mouse Bone. Calcified Tissue International.

[22] Edelman GA, Gally JA (2001). Degeneracy and Complexity in Biological Systems. Proc Natl Acad Sci..

[23] Glatter, O. & Kratky, O. Small Angle A-ray Scattering. *Academic Press* (1982). 10.1016/0076-6879(79)61013-3 481226

[24] Fratzl P, Schreiber S, Boyde A (1996). Characterization of bone mineral crystals in horse radius by small-angle X-ray scattering. Calcified Tissue International.

[25] Poundarik AP (2012). Dilatational Band Formation in Bone. Proc Natl Acad Sci..

[26] Kaartinen MT, Pirhonen A, Linnala-Kankkunen A, Maenpa PH (1999). Cross-linking of Osteopontin by Tissue Transglutaminase Increases Its Collagen Binding Properties. The Journal of Biological Chemistry.

[27] Hauschka PV, Carr SA (1982). Calcium-dependent alpha-helical structure in osteocalcin. Biochemistry.

[28] Francis MD (1969). Inhibition of Calcium Hydroxyapatite Crystal Growth. Calcified Tissue International.

[29] Ritter NM, Farach-Carson MC, Butler WT (1992). Evidence for the formation of a complex between osteopontin and osteocalcin. Journal of Bone and Mineral Research.

[30] Cancela ML, Williamson MK, Price PA (1995). Amino-acid sequence of bone Gla protein from the African clawed toad Xenopus laevis and the fish Sparus aurata. Int. J. Peptide Protein Res..

[31] Reinholt FP, Hultenby K, Oldberg A, Heinegard D (1990). Osteopontin—A possible anchor of osteoclasts to bone. Proc Natl Acad Sci..

[32] Fratzl P (1992). Mineral crystals in calcified tissues: A comparative study by SAXS. Journal of Bone and Mineral Research.

[33] Rinnerthaler S (1999). Scanning Small Angle X-ray Scattering Analysis of Human Bone Sections. Calcified Tissue International.

